# Hedgehog pathway responsiveness correlates with the presence of primary cilia on prostate stromal cells

**DOI:** 10.1186/1471-213X-9-50

**Published:** 2009-10-07

**Authors:** Jingxian Zhang, Robert J Lipinski, Jerry J Gipp, Aubie K Shaw, Wade Bushman

**Affiliations:** 1 Department of Urology, University of Wisconsin-Madison, Madison, WI, USA

## Abstract

**Background:**

Hedgehog (Hh) signaling from the urogenital sinus (UGS) epithelium to the surrounding mesenchyme plays a critical role in regulating ductal formation and growth during prostate development. The primary cilium, a feature of most interphase vertebrate cell types, serves as a required localization domain for Hh signaling transducing proteins.

**Results:**

Immunostaining revealed the presence of primary cilia in mesenchymal cells of the developing prostate. Cell-based assays of a urongenital sinus mesenchymal cell line (UGSM-2) revealed that proliferation-limiting (serum starvation and/or confluence) growth conditions promoted cilia formation and correlated with pathway activation associated with accumulation of Smoothened in primary cilia. The prostate cancer cell lines PC-3, LNCaP, and 22RV1, previously shown to lack demonstrable autocrine Hh signaling capacity, did not exhibit primary cilia even under proliferation-limiting growth conditions.

**Conclusion:**

We conclude that paracrine Hedgehog signaling activity in the prostate is associated with the presence of primary cilia on stromal cells but that a role in autocrine Hh signaling remains speculative.

## Background

The prostate is a multi-lobed male accessory sex gland composed of complex secretory ductal structures that drain into the prostatic urethra. The prostate develops from the prostatic anlagen of the urogenital sinus (UGS) where the hallmark event is budding of UGS epithelium into the surrounding mesenchyme and initiation of ductal growth and morphogenesis. Hedgehog signaling plays a key role in this process, being required for normal budding and ductal outgrowth [1-4]. Of the three vertebrate Hh ligands [Sonic hedgehog (Shh), Indian hedgehog (Ihh) and Desert hedgehog (Dhh)], Shh mRNA is the most abundantly expressed in the developing mouse prostate [4]. Expression in the epithelium of the urogenital sinus (UGS), increases prior to the initiation of ductal budding at embryonic day 17.5 (E17.5) and then localizes to the tips of the nascent ducts [5]. During prostate development Shh appears to act primarily in a paracrine fashion, inducing expression of *Gli1*, *Ptc1*, and other recently identified Hh target genes in the adjacent mesenchyme [[5], unpublished observations]. However, the presence of low *Ptc1*, *Gli1 *and *Gli3 *expression in the urogenital sinus epithelium leaves open the possibility of limited autocrine signaling activity [6]. Hh ligand expression and pathway activity is common in localized prostate cancer (PCa) and may promote tumor cell proliferation by a combination of autocrine and paracrine signaling [7-9] via canonical ligand-mediated signal transduction and/or genetic mutations affecting the regulation of Hh pathway activity in the tumor cells as suggested by Sheng et al., [10]. Hh pathway activity is dramatically increased in advanced, metastatic PCa [8] but whether this represents mutational activation or an increased responsiveness of the tumor cell or ectopic stroma to Hh ligand is not known (For review, see [11]).

Primary non-motile cilia are microtubule-based organelles formed by active interflagellar transport (IFT) present on most vertebrate cells [12]. Recent evidence indicates that the primary cilium is a required cellular feature for canonical vertebrate Hh signal transduction [13]. Hedgehog ligand binding to the 12-pass transmembrane protein Patched relieves its repression of the 7-pass transmembrane protein Smoothened (Smo), followed by accumulation of Smo along with the transcription factors Gli2 and Gli3 in the primary cilium [14,15]. Functional ciliary IFT is required for regulating the activity of Gli2 and Gli3 [16], which coordinately modulate the expression of Hh target genes.

We recently described the generation and characterization of an immortalized cell line (UGSM-2) isolated from the E16.5 UGS mesenchyme [17]. The Hh responsiveness of this cell line was noted to increase with conditions of growth arrest - confluence and serum starvation - conditions associated with the formation of primary cilia. This observation prompted us to examine the expression of primary cilia in the mesenchyme of the developing prostate and to examine the functional requirement for primary cilia in the response of prostate mesenchymal and stromal cells to Hh ligand.

## Methods

### Cell lines

Human prostate myofibroblast WPMY-1 cells were purchased from American Type Culture Collection (ATCC, Manassas, VA) and maintained in recommended media. Mouse prostate mesenchymal UGSM-2 cells were maintained as described previously [17]. For gene expression assays, cells were plated in media containing 10% FBS at 2.0×10^4 ^(subconfluent) or 1×10^5 ^cell/well (confluent) in 400 μl media in a 24-well plate and allowed to attach overnight. Following, cells were washed in media containing 0.1% FBS, which was then replaced with fresh medium containing 10% FBS (high serum) or 0.1% FBS (low serum) ± 1 nM octylated (Curis, Inc., Cambridge, MA) and ± 10 μM cyclopamine (Toronto Research chemicals, Ontario, Candada). Human prostate cancer cell lines were maintained as described previously [18]. For immunocytochemistry assays, cells were plated at 1.0 × 10^4^/well in 4 well chamber slides.

### Plasmids and Retroviral infection

Vectors pLTR-hGli1, pCMV-hGli2β were kindly provided by Dr. Philip Iannaccone (Northwestern University, Chicago, IL) and gDHuSMO-M2 containing cDNA of activated human Smoothened was a generous gift from Genentech, Inc. (South San Francisco, CA). Plasmids containing Myc-tagged mouse wild type Smoothened (WT-Smo) and ciliary localization defective smoothened (CLD-Smo) were kindly provided by Dr. Jeremy Reiter (University of California, San Francisco, CA). Each was subcloned into a retrovirus vector pCMV-IRES-GFP (Gift from Dr. Michael Hoffman, University of Wisconsin, Madison, WI) using standard molecular cloning techniques. All the constructions were confirmed by gel electrophoretic analyses and sequencing. Retroviruses were generated as described [19]. Cells were infected with virus and following one week of passage, GFP+ cells were collected by flow cytometry.

### Immunohistochemistry

Cells grown in four-well chamber slides were fixed in 4% paraformaldehyde for 30 minutes at room temperature. Prostate tissues were isolated from P1 CD-1 mice. Formalin-fixed, paraffin embedded sections were dewaxed, rehydrated, and processed for antigen retrieval. Immunohistochemistry was performed using the following primary antibodies; Rabbit anti-Myc (Abcam, ab9106, 1:200); Mouse anti-acetylated tubulin (Sigma, T6793, 1:1000). The following secondary antibodies were purchased from Molecular Probes and used at 1:200 dilution; Alexa Fluor 546 goat anti- rabbit IgG; Alexa fluor 488 goat anti mouse IgG; Alexa fluor 546 goat anti-mouse IgG. Mouse anti-p63 (Santa Cruz, sc-8431, 1:100) antibody was directly labeled with Alexa Fluor 488 using a monoclonal antibody labeling kit (Invitrogen, A-20181). Slides were mounted with Vectashield Hardset + DAPI mounting media (Vector, Burlingame, CA) and imaged using an Olympus BX51 or BD pathway fluorescent microscope. The tissue sections were imaged using Bio-Rad Radiance 2100 MP Rainbow confocal/multiphoton microscope with LaserSharp software.

### Scanning electron microscopy

Scanning electron microscopy was performed as previously described [20].

### RNA isolation and Real time-PCR

RNA was harvested and cDNA generated as previously described (Lipinski et al., 2006). Gene expression was assayed by Real Time RT-PCR on BioRad iCycler (Hercules, CA) with expression normalized to glyceraldehydes-3-phosphate dehydrogenase (GAPDH). Primer sequences used in this study are listed in Table 1.

**Table 1:** Sequences of real time RT-PCR Primers

**Gene**	**Forward Primer**	**Reverse Primer**
mGAPDH	AGCCTCGTCCCGTAGACAAAAT	CCGTGAGTGGAGTCATACTGGA
mPatched	CTCTGGAGCAGATTTCCAAGG	TGCCGCAGTTCTTTTGAATG
mGli1	GGAAGTCCTATTCACGCCTTGA	CAACCTTCTTGCTCACACATGTAAG
mSmoothened	TTGTGCTCATCACCTTCAGC	TGGCTTGGCATAGCACATAG
hGAPDH	CCACATCGCTCAGACACCAT	GCAACAATATCCACTTACCAGAGTTAA
hPtc1	CGCTGGGACTGCTCCAAGT	GAGTTGTTGCAGCGTTAAAGGAA
hGli1	AATGCTGCCATGGATGCTAGA	GAGTATCAGTAGGTGGGAAGTCCATAT

### Statistical analysis

Data presented is the mean and standard error of three replicate experiments and assessed for significant differences by unpaired t-test. Reported differences have a P-value of ≤ 0.05.

## Results and Discussion

**Figure 1:**
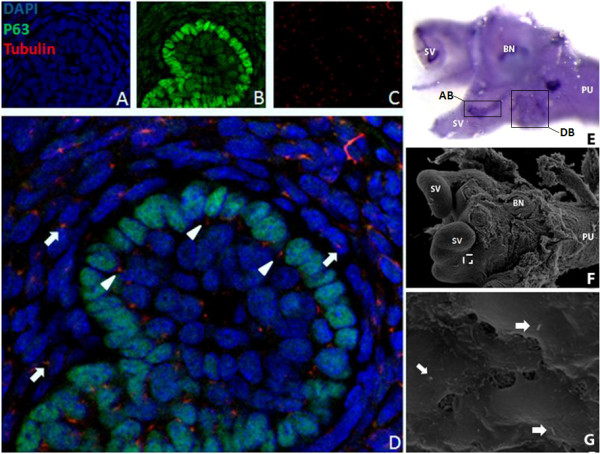
**Stromal and epithelial cells of the newborn prostate feature primary cilia**. Tissue sections of the P1 UGS were costained with DAPI (A), P63, a marker of basal epithelial cells (B), and anti-acetylated tubulin (C). The merged image (D) illustrates that both stromal cells, outside of the ordered ring of basal epithelium cells (arrows), as well as epithelial cells within the basal layer (arrow heads) feature primary cilia. (E) Whole mount in situ hybridization for *Notch1 *of the P1 UGS. Blue staining shows *Notch1*-expressing epithelium (BN, bladder neck; PU, prostatic urethra; SV seminal vesicle). Areas of anterior (AP) and dorsal (DP) prostate budding are indicated. (F) Scanning electron micrograph of the P1 male UGS. (G) High magnification image of the boxed area in F showing cilia on the surface mesenchyme (arrows).

*In situ *hybridization assays have demonstrated epithelial expression of *Sonic Hedgehog *(*Shh*) and *Indian Hedgehog *(*Ihh*) in the developing prostate and robust expression of the conserved Hh target genes *Ptc1 *and *Gli1 *in mesenchyme of the mouse urogenital sinus [4,5]. Using immunohistochemical staining for acetylated tubulin, we demonstrate the presence of primary cilia on mesenchymal cells surrounding the nascent ducts of the newborn (P1) mouse prostate (Figure 1). Staining also revealed the presence of cilia on epithelial cells in the ductal buds. The presence of cilia on the mesenchymal cells was verified by scanning electron microscopy. Imaged mesenchymal cells overly the region of anterior budding, as demonstrated by staining for *Notch1 *which marks epithelial buds [[21], unpublished observations] in a comparable sample.

**Figure 2:**
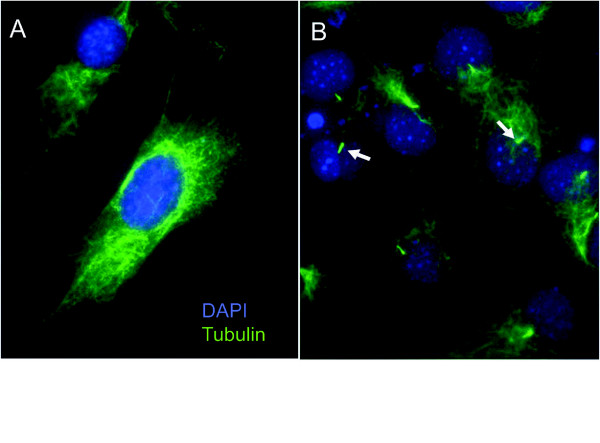
**Cilia presence in urogenital sinus mesenchyme cells is dependent on proliferation regulating growth conditions**. UGSM-2 cells were plated at subconfluence and grown in high serum (10% FBS) (A) and low serum (0.1% FBS) (B) media. Nuclei are stained with DAPI (blue), cilia and microtubules are stained with anti-acetylated tubulin (green).

**Figure 3:**
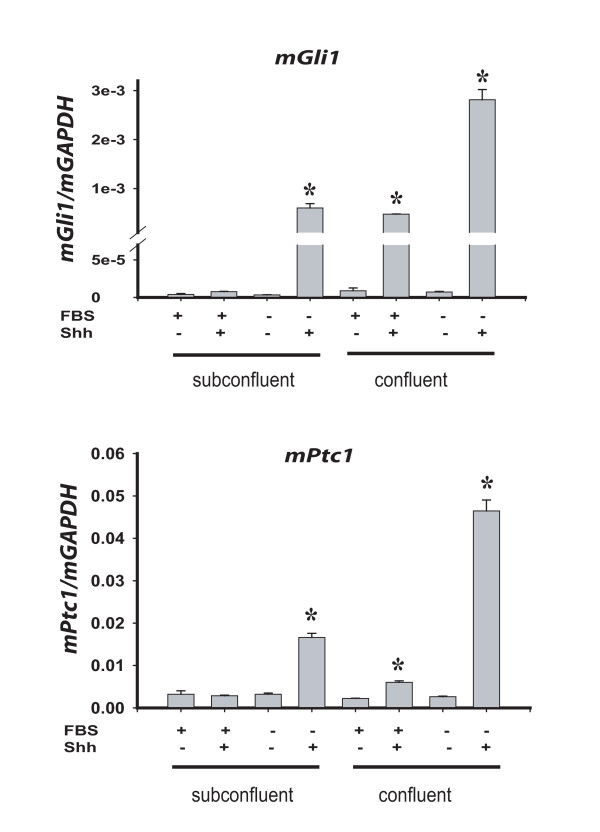
**Cilia presence correlates with Hh signaling capacity in urogenital sinus mesenchyme cells**. UGSM-2 cells were plated at confluence or subconfluence and grown in 0.1% FBS (FBS-) or 10% FBS (FBS+) media. Following 48 hrs incubation +/- 1nM Shh peptide, RNA was isolated and gene expression was determined by Real Time RT-PCR. Data represents the mean ± standard error of three replicate experiments. * indicates p < 0.05.

To test whether primary cilia promote Hh signal transduction in UGS mesenchymal cells, we examined the correlation of primary cilia with Hh responsiveness in the immortalized mouse urogenital sinus mesenchymal cell line (UGSM-2). Because primary cilia are a feature of cells at interphase, we compared cells under high proliferative growth conditions (subconfluence and high serum [10% FBS]) versus limited proliferative growth conditions (low serum [0.1%] and/or cellular confluence) [22]. Immunohistochemical staining for acetylated tubulin revealed that UGSM-2 cells grown at subclonfluence in high serum media largely lacked cilia, whereas a majority of cells at confluence and/or in low serum featured prominent primary cilia (Figure 2). We found that subconfluent proliferating UGSM-2 cells largely lacking cilia also demonstrated a muted a transcriptional response to Hh ligand stimulation (Figure 3). However, cilia-expressing UGSM-2 cells grown in low serum exhibited a robust ligand-stimulated induction of Hh target genes, which was augmented by cellular confluence. These observations correlate the appearance of primary cilia under conditions of limited proliferation with transcriptional responsiveness to Hh-ligand and provide an explanation for the previous report that Hh responsive fibroblasts exhibit increasing responsiveness to Hh ligand with greater confluence [23].

**Figure 4:**
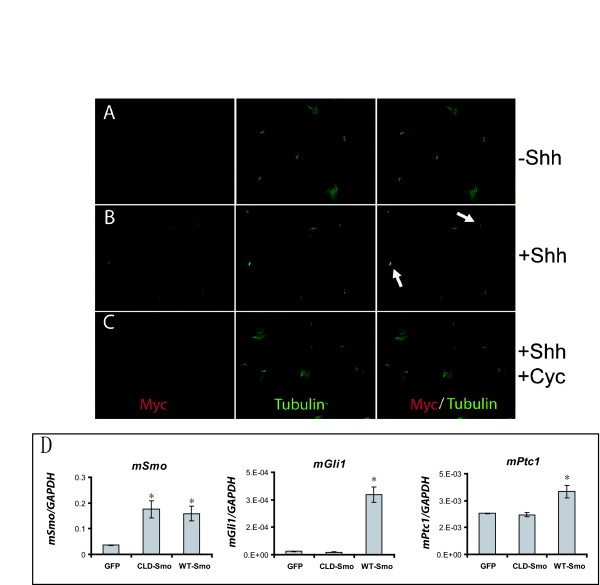
**Hh stimulation triggers Smo localization to the primary cilium**. (A-C) UGSM-2 cells overexpressing myc-tagged Smo were plated at subconfluence and grown in low serum media +/- 1 nM Shh peptide, +/- 10 μM cyclopamine. Cilia and microtubules are stained with anti-acetylated tubulin (green) and Smo-myc with anti-myc (red). Cyan coloring (arrows) indicates ciliary localization of Smo that is not evident in the absence of Shh ligand or in the presence of cyclopamine. (D) UGSM-2 cells overexpressing wild type Smo (WT-Smo) or ciliary localization defective Smo (CLD-Smo) were plated at confluence and grown in low serum media +/- 1 nM Shh peptide. Following 48 hrs, RNA was isolated and gene expression was determined by Real Time RT-PCR. Data represents the mean ± standard error of three replicate experiments. * indicates p < 0.05.

Cyclopamine is a potent chemical antagonist of Hh signaling that binds to Smo and prevents Hh ligand-induced localization to the primary cilium [15]. Employing immunocytochemistry for myc-tagged Smoothened [15], we found that addition of Shh peptide to UGSM-2 cells grown under limited proliferative conditions triggered a localization of exogenous wild type Smo to the primary cilium and that this localization could be blocked by cyclopamine (Figure 4). In contrast, Shh peptide did not trigger localization to the cilia when cells expressed a ciliary localization domain mutated form of Smo (CLD-Smo) [15] (4b). Gene expression analysis demonstrated that while overexpression of wild type Smo was sufficient to induce the expression of Hh target genes *Gli1 *and *Ptc1*, overexpression of CLD-Smo had no effect.

**Figure 5:**
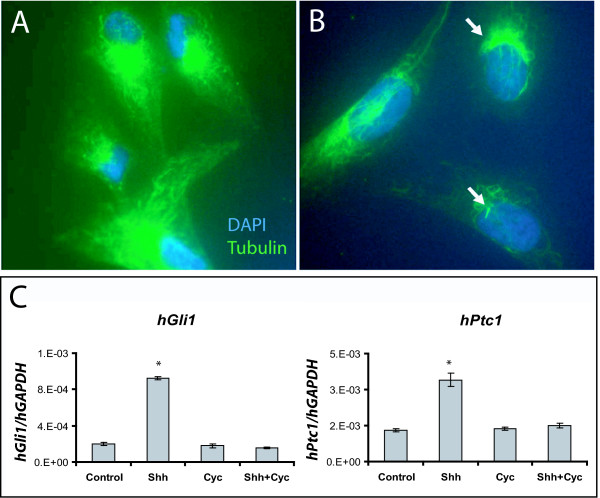
**Cilia presence correlates with Hh signaling capacity in human prostate mesenchymal cells**. Human prostate myofibroblast (WPMY-1) cells were plated at subconfluence and grown in high (A) or low (B) serum conditions for 48 hrs. Nuclei are stained with DAPI (blue), cilia and microtubules are stained with anti-acetylated tubulin (green). (C) WPMY-1 cells were plated at confluence in low serum media +/- 1 nM Shh peptide, +/- 10 μM cyclopamine for 48 hrs. Following, RNA was isolated and gene expression was determined by Real Time RT-PCR. Data represents the mean ± standard error of three replicate experiments. * indicates p < 0.05.

To determine whether primary cilia play an equally critical role in paracrine signaling in the human prostate, we tested the correlation of cilia presence and Hh signaling capacity in a human prostate myofibroblast cell line (WPMY-1). WPMY-1 cells exhibited cilia when grown under limited-proliferative conditions but not when grown under conditions promoting proliferation (Figure 5A, B). And, like UGSM-2 cells, WPMY-1 cells demonstrated transcriptional responsiveness to Shh ligand when grown under proliferation-limiting conditions and this response was inhibited by cyclopamine (Figure 5C).

**Figure 6:**
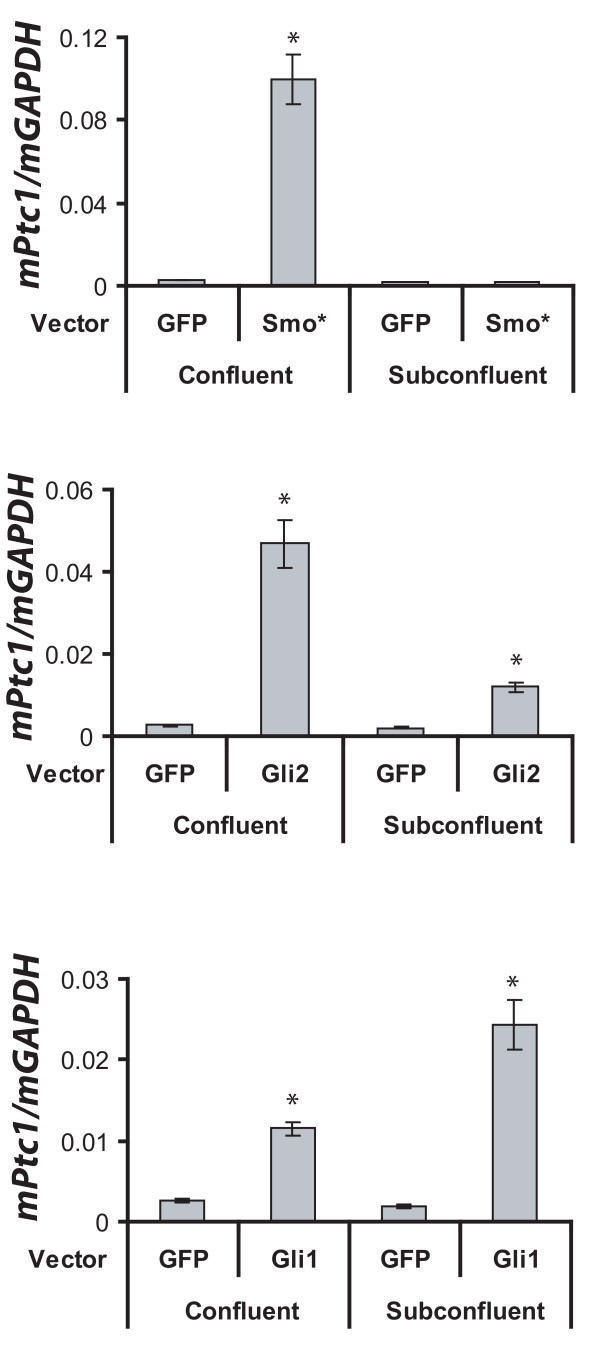
**Growth conditions promoting cilia formation are required for Hh signaling activation by Smo but not Gli1 or Gli2**. UGSM-2 cells overexpressing hGli1-GFP, hGli2-GFP, hSmo-M2, or GFP alone were plated at subconfluence or confluence and grown in low or high serum media. Following 48 hrs, RNA was harvested and gene expression determined by Real Time RT-PCR. Data represents the mean ± standard error of three replicate experiments. * indicates p < 0.05.

Analysis of tissues derived from transgenic mice bearing hypomorphic or null alleles for genes necessary for the formation and function of ciliary machinery including *IFT172*, *Tg737*, and *Kif3a*, have begun to elucidate the role of cilia function in facilitating Hh signal transduction [16,24,25]. Together, these studies demonstrate that Hh ligand stimulation promotes the localization of Smo, Gli2, and Gli3 to the primary cilia that is required for subsequent transcriptional activation and repression activities of Gli2 and Gli3 respectively. These studies predict a differential dependence on the primary cilia for Hh target gene activation by Gli1, on the one hand, and Smo and Gli2 on the other. Indeed, we found that overexpression of Gli1 was sufficient to activate Hh target genes in proliferating cells lacking cilia as well as in proliferation-limited, cilia-bearing cells (Figure 6). In contrast, overexpression of an activated form of Smo was sufficient to activate Hh target gene expression only in cells bearing cilia. While overexpression of full length Gli2 caused only minimal target gene activation in proliferating cells, a robust response was seen in proliferation-limited, cilia expressing cells. This may infer that cilia presence is required for optimal Gli2 function.

**Figure 7:**
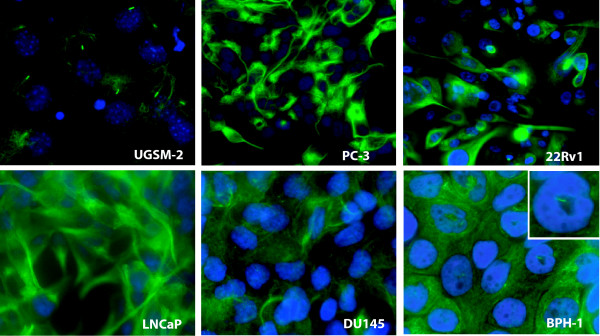
**PC-3, 22RV1, LNCaP, and DU145 prostate cancer cell lines do not express cilia**. Cells were plated at confluence and grown in low serum media. Nuclei are stained with DAPI (blue) and microtublules are stained with anti-acetylated tubulin (green).

Several commonly used prostate cancer cell lines have been reported to be Hh-responsive [8,9] but this remains controversial. Our previous studies have shown that the LNCaP, PC3 and 22RV1 cell lines fail to exhibit transcription of the canonical Hh target genes *Ptc1 *or *Gli1 *when treated with either Shh ligand or transfected with activated Smo [18]. Using several methodologies, McCarthy and Brown [26] also found no evidence for autocrine Hh signaling in PC3 cells. Yauch et al. [27], found no evidence for cell-autonomous Hh signaling in a variety of cancer cell lines previously reported to demonstrate autocrine signaling. When we examined the LNCaP, PC-3, DU145, and 22Rv1 cell lines under a variety of growth conditions including confluence and low serum, we found no evidence of cilia formation (Figure 7). An explanation for this could be the inability of cancer cells to undergo growth arrest. The BPH-1 cell line, made by transfection of a benign human epithelial cell with large-T antigen, does exhibit cilia specifically under conditions of confluence and serum starvation, although the cilia appear stunted in comparison to those in UGSM-2 cells. Interestingly, BPH-1 cells appear to be completely unresponsive to Shh ligand even under conditions of growth arrest (unpublished data).

We have shown previously that overexpression of an activated form of Gli2 activates Hh target genes in LNCaP and PC-3 cells, while overexpression of an activated form of Smo does not [18]. These data suggest that a defect in the pathway between Smo and the Gli transcription factors exists in both cell lines, Our finding here that these cells do not exhibit cilia when grown *in vitro *suggests a plausible mechanism for the defect in signal transduction but further investigation will be required to substantiate that paradigm. Future work assessing the role of autocrine Hh signaling in these widely-used cancer cell lines should consider the apparent essential role of cilia in Hh signal transduction and *in vitro *and *in vivo *growth conditions that may affect cilia formation.

## Conclusion

These studies demonstrate the presence of cilia in both epithelial and mesenchymal cells of the developing prostate. As we found that *in vitro *culture conditions of UGSM-2 cells which promote ciliary formation enable functional Hh signal transduction, our observation that cilia are a feature of peri-ductal mesenchyme cells is consistent with the previously described paracrine signaling paradigm where Hh ligand secreted by prostate epithelium acts on adjacent mesenchymal cells and a previous report identifying cilia as a feature of human prostate stromal cells [28]. To our knowledge, work investigating how serum and density conditions of cells grown *in vitro *correlate with the *in vivo *microenvironment of the developing prostate has not been presented and should be an area of future study given these findings. The absence of cilia on several prostate cancer cell lines and a correlative lack of Hh-responsiveness further argues against a role for cell-autonomous Hh signaling and plausibly explains the observation that pathway activation can be achieved by expressing activated *Gli2 *but not activated *Smo*. A role for autocrine signaling in cilia-expressing epithelial cells remains uncertain.

## Authors' contributions

J.Z. participated in designing experiments, was primarily responsible for experiment execution, and participated in manuscript preparation. R.J.L. participated in experimental design and interpretation and wrote the manuscript. J.G. participated in experimental design and manuscript preparation. A.K.S. prepared retroviral constructs. W.B. conceived experimental approach and participated in manuscript preparation. All authors have read and approved the final manuscript.

## References

[B1] Podlasek CA, Barnett DH, Clemens JQ, Bak PM, Bushman W (1999). Prostate development requires Sonic hedgehog expressed by the urogenital sinus epithelium. Dev Biol.

[B2] Freestone SH, Marker P, Grace OC, Tomlinson DC, Cunha GR, Harnden P, Thomson AA (2003). Sonic hedgehog regulates prostatic growth and epithelial differentiation. Dev Biol.

[B3] Wang BE, Shou J, Ross S, Koeppen H, De Sauvage FJ, Gao WQ (2003). Inhibition of epithelial ductal branching in the prostate by sonic hedgehog is indirectly mediated by stromal cells. J Biol Chem.

[B4] Doles J, Cook C, Shi X, Valosky J, Lipinski R, Bushman W (2006). Functional compensation in Hedgehog signaling during mouse prostate development. Dev Biol.

[B5] Lamm ML, Catbagan WS, Laciak RJ, Barnett DH, Hebner CM, Gaffield W, Walterhouse D, Iannaccone P, Bushman W (2002). Sonic hedgehog activates mesenchymal Gli1 expression during prostate ductal bud formation. Dev Biol.

[B6] Pu Y, Huang L, Prins GS (2004). Sonic hedgehog-patched Gli signaling in the developing rat prostate gland: lobe-specific suppression by neonatal estrogens reduces ductal growth and branching. Dev Biol.

[B7] Fan L, Pepicelli CV, Dibble CC, Catbagan W, Zarycki JL, Laciak R, Gipp J, Shaw A, Lamm ML, Munoz A, Lipinski R, Thrasher JB, Bushman W (2004). Hedgehog signaling promotes prostate xenograft tumor growth. Endocrinology.

[B8] Karhadkar SS, Bova GS, Abdallah N, Dhara S, Gardner D, Maitra A, Isaacs JT, Berman DM, Beachy PA (2004). Hedgehog signalling in prostate regeneration, neoplasia and metastasis. Nature.

[B9] Sanchez P, Hernandez AM, Stecca B, Kahler AJ, DeGueme AM, Barrett A, Beyna M, Datta MW, Datta S, Ruiz i Altaba A (2004). Inhibition of prostate cancer proliferation by interference with SONIC HEDGEHOG-GLI1 signaling. Proc Natl Acad Sci USA.

[B10] Sheng T, Li C, Zhang X, Chi S, He N, Chen K, McCormick F, Gatalica Z, Xie J (2004). Activation of the hedgehog pathway in advanced prostate cancer. Mol Cancer.

[B11] Shaw A, Bushman W (2007). Hedgehog signaling in the prostate. J Urol.

[B12] Satir P, Christensen ST (2007). Overview of structure and function of mammalian cilia. Annu Rev Physiol.

[B13] Huangfu D, Liu A, Rakeman AS, Murcia NS, Niswander L, Anderson KV (2003). Hedgehog signalling in the mouse requires intraflagellar transport proteins. Nature.

[B14] Haycraft CJ, Banizs B, Aydin-Son Y, Zhang Q, Michaud EJ, Yoder BK (2005). Gli2 and Gli3 localize to cilia and require the intraflagellar transport protein polaris for processing and function. PLoS Genet.

[B15] Corbit KC, Aanstad P, Singla V, Norman AR, Stainier DY, Reiter JF (2005). Vertebrate Smoothened functions at the primary cilium. Nature.

[B16] Liu A, Wang B, Niswander LA (2005). Mouse intraflagellar transport proteins regulate both the activator and repressor functions of Gli transcription factors. Development.

[B17] Shaw A, Papadopoulos J, Johnson C, Bushman W (2006). Isolation and characterization of an immortalized mouse urogenital sinus mesenchyme cell line. Prostate.

[B18] Zhang J, Lipinski R, Shaw A, Gipp J, Bushman W (2007). Lack of demonstrable autocrine hedgehog signaling in human prostate cancer cell lines. J Urol.

[B19] Cui Q, Lim SK, Zhao B, Hoffman MF (2005). Selective inhibition of TGF-beta responsive genes by Smad-interacting peptide aptamers from FoxH1, Lef1 and CBP. Oncogene.

[B20] Dunty WC, Zucker RM, Sulik KK (2002). Hindbrain and cranial nerve dysmorphogenesis result from acute maternal ethanol administration. Dev Neurosci.

[B21] Shou J, Ross R, Koeppen H, de Sauvage FJ, Gao W (2001). Dynamics of Notch expression during murine prostate development and tumorigenesis. Cancer Res.

[B22] Wheatley DN, Wang AM, Strugnell GE (1996). Expression of primary cilia in mammalian cells. Cell Biol Int.

[B23] Taipale J, Chen JK, Cooper MK, Wang B, Mann RK, Milenkovic L, Scott MP, Beachy PA (2000). Effects of oncogenic mutations in Smoothened and Patched can be reversed by cyclopamine. Nature.

[B24] Zhang Q, Davenport JR, Croyle MJ, Haycraft CJ, Yoder BK (2005). Disruption of IFT results in both exocrine and endocrine abnormalities in the pancreas of Tg737(orpk) mutant mice. Lab Invest.

[B25] Ocbina PJ, Anderson KV (2008). Intraflagellar transport, cilia, and mammalian Hedgehog signaling: analysis in mouse embryonic fibroblasts. Dev Dyn.

[B26] McCarthy FR, Brown AJ (2008). Autonomous Hedgehog signalling is undetectable in PC-3 prostate cancer cells. Biochem Biophys Res Commun.

[B27] Yauch RL, Gould SE, Scales SJ, Tang T, Tian H, Ahn CP, Marshall D, Fu L, Januario T, Kallop D, Nannini-Pepe M, Kotkow K, Marsters JC, Rubin LL, de Sauvage FJ (2008). A paracrine requirement for hedgehog signalling in cancer. Nature.

[B28] Planz B, Kirley SD, Wang Q, Tabatabaei S, Aretz HT, McDougal WS (1999). Characterization of a stromal cell model of the human benign and malignant prostate from explant culture. J Urol.

